# Pre-pregnancy stress induces maternal vascular dysfunction during pregnancy and postpartum

**DOI:** 10.1007/s43032-023-01248-2

**Published:** 2023-05-23

**Authors:** Mary Gemmel O’Donnell, Lauren Stumpp, Marcia J. Gallaher, Robert W. Powers

**Affiliations:** 1 Magee-Womens Research Institute, Pittsburgh, PA 15213 USA; 2 Department of Obstetrics, Gynecology and Reproductive Sciences, University of Pittsburgh School of Medicine, Pittsburgh, PA 15213 USA; 3 Department of Biology, Thiel College, Greenville, PA 16125 USA

**Keywords:** Mental health, Anxiety, Depression, Pregnancy, Vascular function

## Abstract

An estimated 20% of women suffer from a stress-related mood disorder including depression and anxiety during and after pregnancy, making these disorders among the most common complications of pregnancy. These stress-related disorders are associated with adverse pregnancy outcomes including gestational hypertension and preeclampsia, which are associated with poor cardiometabolic health postpartum. Despite these associations, the direct impact of stress and related disorders on maternal vascular health, and contributing mechanisms, remain understudied. The aim of this study was to investigate the effect of pre-pregnancy stress on maternal vascular outcomes in a BALB/c mouse model of chronic unpredictable stress. Maternal blood pressure and ex-vivo vascular function were investigated during pregnancy and postpartum. Offspring characteristics were assessed at the end of pregnancy and postpartum. Main findings show that pre-pregnancy stress exposure increased blood pressure during mid and late pregnancy and impaired ex vivo vascular function at the end of pregnancy. These effects persisted into the postpartum period, suggesting a long-term effect of stress on maternal vascular health, which appear to be partially attributable to disruptions in nitric oxide (NO) pathway signaling. These data suggest exposure to stress and related disorders, even prior to pregnancy, can contribute to vascular complications during pregnancy and postpartum.

## Introduction

An estimated 20% of women suffer from stress-related disorders during pregnancy [1–4]. This makes stress-related disorders, such as anxiety and depression, among the most common during pregnancy and postpartum. Further, the prevalence of these conditions do not account for the recent impact of the COVID-19 pandemic on perinatal stress [5–7], and therefore current estimates of stress-related illness during pregnancy are likely underrepresented. Due to the association between stressful life events, stress related disorders, and poor pregnancy outcome [8, 9], more work is needed to understand how stress affects maternal and infant outcomes during pregnancy and postpartum.

Previous research has shown a transgenerational association between perinatal stress and offspring outcome. Stress-related disorders contribute to poor cognitive function, aberrations in neurodevelopment and neurotransmitter levels, altered synaptic plasticity, changes in the hypothalamic–pituitary–adrenal (HPA)-axis, and behavioral changes in offspring [10–13]. Further, stress exposure alters the trajectory of development in offspring, resulting in persistent changes in adult neurobehavior compared to non-stressed offspring [10–13]. However, most research has focused on offspring outcome alone, with minimal work investigating how stress and related disorders impact maternal well-being and long-term health. Preliminary work has associated perinatal stress and related disorders with altered neuroendocrine function and comorbidities including gestational hypertension and preeclampsia [14–16]. Therefore, as one of the most common risk factors affecting pregnant women and a significant contributor to perinatal comorbidities, more work is needed to understand the impact of stress-related disorders on systemic maternal health during pregnancy.

Due to the association of stress-related disorders with pregnancy related co-morbidities [14–18], pregnancy may serve as a unique time to identify women at risk for future vascular related disorders. Clinically, perinatal depression has been associated with hypertensive disorders of pregnancy, including pre-eclampsia and gestational hypertension, and is associated with an increased prevalence of cardiomyopathy [14–16, 19]. Poor vascular health and prevalence of hypertensive conditions during pregnancy is associated with an increase in cardiovascular disease risk throughout life [20, 21]. Women suffering from maternal mood disorders may therefore be more susceptible to poor cardiovascular health during pregnancy, which may prime individuals for future cardiovascular disorders. Since heart disease is the leading cause of death among women in the USA, early identification of women at risk for cardiovascular disease postpartum is an important step to improve maternal health. Therefore, more work is needed in rodents to identify underlying mechanisms contributing to poor vascular adaptations in pregnancies challenged by perinatal stress-related mood disorders.

The chronic unpredictable stress (CUS) paradigm has been shown to induce anxiety-like behavior and alter serotonin metabolism during the peripartum period [17, 18], mimicking the clinical associations between stress, depression, and anxiety. Further, exposure to stress results in persistent, long-lasting changes to behavior and neuroendocrine response in rodents which mimics clinical symptomology of stress-related disorders [22–24]. This model therefore serves as a unique resource to investigate the impact of stress and stress-related disorders on vascular function during pregnancy and postpartum. The aim of this study was to first, investigate the impact of pre-pregnancy stress on vascular function during pregnancy using the CUS model. In the present study, open field testing was used to measure the impact of perinatal CUS on behavioral phenotypes. In addition, measures of maternal vascular health were assessed during pregnancy by investigating maternal blood pressure and ex vivo vascular function. Additional measures of pregnancy health including pregnancy success, maternal heart weight, placental histology, and offspring characteristics were measured. In addition, this study aimed to investigate the possible long-term impact of perinatal stress on maternal vascular health. Lastly, we investigated the effect of pre-pregnancy stress on maternal blood pressure, ex vivo vascular function, and heart weight in the postpartum period.

## Methods

### Animals

Prior to breeding, eight-week old BALB/c female and male mice from Jackson Labs (Bar Harbor, ME) were multi-housed in a temperature-controlled room with 12-:12-h light/dark cycle and ad libitum access to standard chow. The BALB/c strain was selected as research has documented increased susceptibility to stressors, and more distinct anxiety-like phenotypes, in this strain compared with other mouse strains [25, 26]. All experiments were approved by the Institutional Animal Care and Use Committees of Magee-Womens Research Institute and the University of Pittsburgh, Pittsburgh, PA, USA.

A model of chronic unpredictable stress (CUS) prior to breeding was utilized as it induces aspects of stress-related mood disorders during pregnancy and postpartum via increased depressive/anxiety-like behavior and alterations in neuroendocrine measures [18, 27–30], without genetic or pharmacological manipulation. Pre-pregnancy stress exposure was used as the neurobehavioral impact of stress paradigms is often observable after the exposure itself. For example, perinatal stress exposure results in an altered neurobehavioral phenotype postpartum [31, 32]. Therefore, pre-pregnancy exposure mimics stress-related disorders during pregnancy. Further, published data indicates women at greatest risk of developing perinatal stress-related mood disorders are those with a history of prolonged stress and stressful life events [16, 33]. Prior to breeding, female mice were randomly assigned to stress (n = 22) or control (no stress, n = 22) conditions, and mice in the CUS group were exposed to 3 weeks of randomized stressors. Stressors, applied up to 2 times per day for the 3-week exposure period, consisted of light exposure in shallow bath (45 min), movement of cage location (12 h), damp bedding (8 h), food deprivation (6 h), cage tilt (6 h) and forced swimming (5 min) [18, 25, 34–37]. All stressors involving water (light exposure in shallow bath, damp bedding, forced swimming) utilized 30 °C water and towel-drying of animals following exposure. Following stress, CUS-exposed animals were singly housed to serve as a persistent stressor [38]. Non-stressed animals were multi-housed in accordance with IACUC policies. Prior to breeding, all animals were subjected to open field testing (detailed below) to assess anxiety and depressive-like behavior. Immediately following behavioral testing, breeding procedures occurred.

For breeding, time mating included afternoon placement of female mice in male cages with the presence of a copulation plug on the following morning identifying gestation day (GD) 0.5. In the event of a lost pregnancy, rebreeding took place on the day following pre-pregnancy weight observation. In accordance with IACUC policies, all pregnant and post-partum females were singly-housed. Females from both groups were assessed either during pregnancy (GD 17.5) or at 1 month postpartum. For both groups, and at each time point, blood pressure was measured by tail cuff. On experiment day, mice were weighed and euthanized via carbon dioxide asphyxiation and cervical dislocation. Placental and heart tissue was immediately excised and weighed and small mesenteric arteries, representative of the microvasculature, were dissected for assessment of ex-vivo vascular function by wire myography. Pregnancy data included collection of weekly weights which were compared to the previous week to assess percent weight gain. Pregnancy data also included pup and placental data. Postpartum experiments included collection of maternal behavior data, repeated open field testing, and offspring data. For postpartum offspring data, 2 females and 2 males were assessed at postpartum day 28 to serve as representative data points.

### Open Field Testing

Activity in an open field arena was completed immediately prior to breeding to assess anxiety-like behavior. Prior to testing, animals were habituated to a darkened polyurethane apparatus (50 cm × 50 cm × 50 cm) for 15 min. On the following day, females were placed in the center of the arena and behavior was recorded by an overhead mounted digital camera for 10 min. Videos were coded, randomized and evaluated by a blinded observer using JWatcher software. Scored behaviors included time and number of bouts spent in peripheral and central zones of the arena as well as freezing and rearing episodes. Time to first movement was also recorded. In the postpartum period, open field testing (OFT) was repeated prior to experiment day to quantify the persistent effect of stress on anxiety-like behavior.

### Blood Pressure

To assess in vivo vascular function, blood pressure was measured by CODA-2 volume-pressure recording tail-cuff monitoring system (Kent Scientific, Torrington, CT) [39]. Measurements were taken during mid (GD9.5–10.5) and late (GD 14.5–17.5) pregnancy, or at 1 month postpartum. Prior to data collection, mice were habituated to a warmed cylindrical holder and CODA measurement protocol for 20 min per day for 2 days. On the day of assessment, following 10 acclimation cycles, systolic (SBP) and diastolic (DBP) blood pressures were obtained by averaging 5 valid cycles of the 10-cycle measurement period. Mean arterial pressure (MAP) was calculated by (SBP + 2*DBP)/3.

### Maternal Behavior

To quantify maternal caregiving behavior, maternal behavior was observed on postnatal day (PD) 1- PD6 two times per day for 5 min based on previous literature [18]. Time and number of bouts in the following maternal behaviors were recorded: licking (licking/grooming, licking while on the nest); nursing (blanket nursing, and/or passive nursing); nest building and time off the pups. Maternal self-grooming behavior was also assessed.

### Vascular Function

Ex vivo endothelial-dependent and -independent vascular function was measured using a multiwire four chamber myograph system from DMT (Danish Myo Technology, Denmark) [39]. Second order mesenteric arteries were dissected and mounted with 10-µm wires in a 7 mL organ bath of HEPES-buffered saline at 37 °C (142 mmol/L NaCl, 4.7 mmol/L KCl, 1.18 mmol/L KH2PO4, 1.17 mmol/L MgSO4 7H2O, 2.5 mmol/L CaCl2 2H2O, 10 mmol/L HEPES, and 5.5 mmol/L dextrose, pH 7.4). Following 20 min of equilibration, arteries were subjected to a pre-conditioning stretch of 1.8mN. Optimal tension was obtained when arteries were set at 85% of the calculated arterial internal circumference (using the Law of LaPlace) that would be obtained if the artery was at 100 mmHg (0.85L_100_). Subsequent dose response curves were generated by cumulative addition of adrenergic agonist phenylephrine (10^−7^ to 10^−5^ mol/L) to assess vessel contraction. In the same vessels, following 50% maximum contraction by phenylephrine, endothelial-dependent and -independent vasodilation and subsequent relaxation was assessed by addition of methacholine (10^−10^ to 10^−5^ mol/L) and sodium nitroprusside (10^−10^ to 3 × 10^−7^ mol/L), respectively. The response to methacholine and nitroprusside are expressed as the “percent contraction remaining” in which 100% is the initial stable contraction to the EC50 of phenylephrine. To assess nitric oxide (NO) mediated vascular function, dose response curves to phenylephrine and methacholine were repeated following 20-min pre-incubations in 10^−4^ mol/L NG-nitro-L-arginine methyl ester (L-NAME) to inhibit NO synthase. Inclusion of L-NAME allowed for investigation of the effect of CUS on the NO signaling pathway. Prior to each dose response, arteries were washed with HEPES buffer and allowed to re-equilibrate for 20 min. All chemicals were purchased from Sigma (St. Louis, MO) and each dose response is reported as a percent of total contraction. Change in relaxation response to methacholine and methacholine in the presence of L-NAME (delta-methacholine) was calculated by: (% contraction remaining to methacholine + L-NAME)—(% contraction remaining to methacholine) for each dose. Vascular function experiments serve as a terminal endpoint and therefore data presented are cross-sectional.

### Histology

Placental tissue was collected immediately after euthanasia and immersion fixed in 4% paraformaldehyde for processing by histology core. Following fixation, tissue was frozen in O.C.T. compound and sliced in 4 micron sections by cryostat (Leica Biosystems, Wetzlar, Germany) for hemotoxylin and eosin staining (Surgipath, Leica Biosystems). Paraffin-embedded slides were deparaffinized, re-hydrated with distilled H_2_O, and stained with hematoxylin for 5 min. Sections were sequentially washed with H_2_O and incubated for 1 min in Define and Blue Buffer. Slides were washed in 70% EtOH and counterstained with eosin with phloxine. Following counterstain, slides were dehydrated, mounted, and allowed to dry prior to quantification. Stained placental sections were scanned using a Nikon 90i microscope and Nikon DS camera with Nikon NIS Element Software (Nikon, Minato, Tokyo, Japan) under a 4 × objective to obtain whole-section photomicrographs. For each animal, eight stained sections per placenta were quantified by blinded observer using Image J software (Wayne Rasband, NIH, Bethesda MD, USA). Decidual, junctional-zone, and labryinth length and area were quantified for each section, and normalized to respective placental length and areas [40–42]. Histological measurements for each animal were averaged and used for statistical analysis.

### Statistical Analysis

Analyses were performed using Statistica software (Dell Inc.). Data distributions were evaluated for normality, unpaired independent two-sample student’s t-tests were used to compare groups, and summary data are presented as mean ± standard error. For comparisons of litter characteristics, ANCOVAs were used to assess pup weight, placental weight, and pup to placental ratio with litter size as a covariate. ANCOVAs were also performed to ensure that female age at breeding was not a significant covariate in the present study. *P*-values < 0.05 were considered significant. Figure legends and tables delineate sample sizes per group per timepoint.

## Results

### Pregnancy

#### Pre-pregnancy Stress Induced Anxiety-Like Behavior

**Fig. 1:**
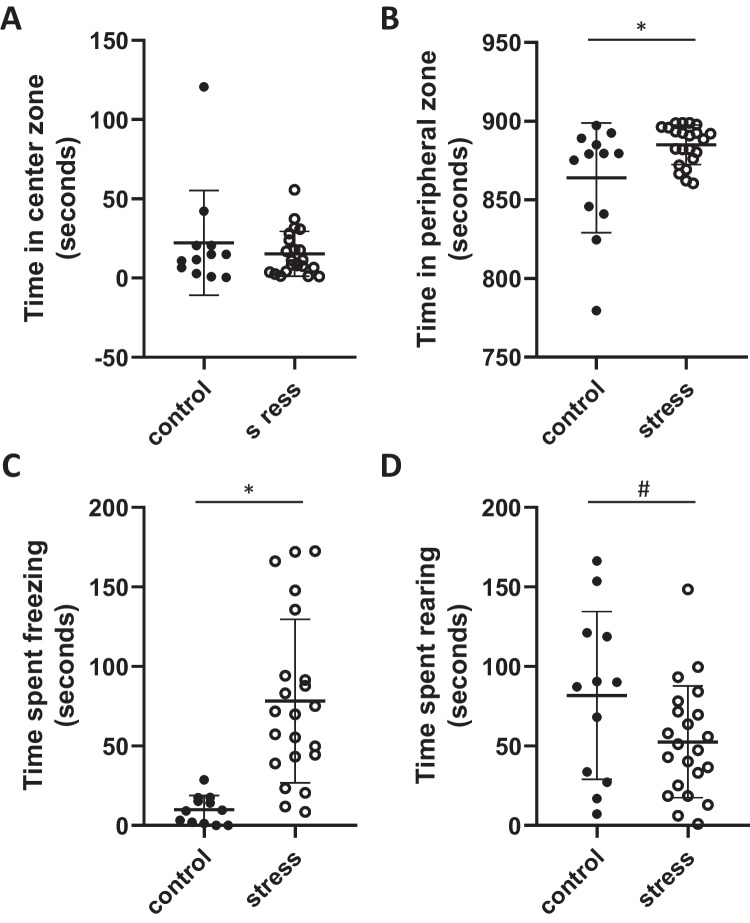
Open field behavior. Mean (± SD) time spent **A** in the center zone, **B** in the peripheral zone, **C** freezing and **D** rearing in the open field arena. Stress exposure increased **B** time in the peripheral zone, **C** time spent freezing, and **D** decreased rearing (exploratory) behavior in the open field arena when compared to control females. * denotes significant difference (p < 0.05), # denotes trend (*p* = 0.06) (control *n* = 12, stress *n* = 22)

Exposure to stress did not impact time spent in the center zone of the arena (*p* = 0.3, Fig. 1A) but did significantly increase time spent in the peripheral zone of the open field arena (*p* = 0.02, *T* = 2.6, *F* = 7.8, df = 32, Fig. 1B), indicating increased anxiety-like behavior. Females exposed to stress also spent significantly more time freezing in the open field test (*p* < 0.01, *T* = 4.5, *F* = 32.5, df = 32, Fig. 1C), and displayed an increased number of freezing bouts (*p* < 0.01, *T* = 5.9, *F* = 10.8, df = 32, Table 1) compared to control females, supporting increased anxiety-like behavior in CUS-exposed females. Further, there was a trend toward stress exposure decreasing time spent rearing (exploring) in the open field test compared to control females (*p* = 0.06, Fig. 1D), although this did not reach significance and there was no difference in the number of rearing bouts between groups (*p* = 0.8, Table 1). There was no effect of stress exposure on time to first movement in the open field test (*p* = 0.1, Table 1), number of entries into the periphery (*p* = 0.8, Table 1), nor number of entries into the center zone of the arena (*p* = 0.8, Table 1). No difference in peripheral or central bouts suggests differences in open field behavior were indeed anxiety-related as compared to locomotion-related.

**Table 1:** Open field behavior in stress-exposed and control females

	Control (*n* = 12)	Stress (*n* = 22)
Time to first movement (seconds)	1.3 ± 1.0	2.3 ± 1.9
Peripheral entries	12 ± 9	11 ± 8
Bouts freezing	8 ± 5	41 ± 19*
Bouts rearing	53 ± 30	50 ± 32
Central zone entries	11 ± 9	10 ± 9

#### Pre-pregnancy Stress Decreased Early Pregnancy Weight Gain and Increased Blood Pressure and Heart Weight During Late Pregnancy

**Fig. 2:**
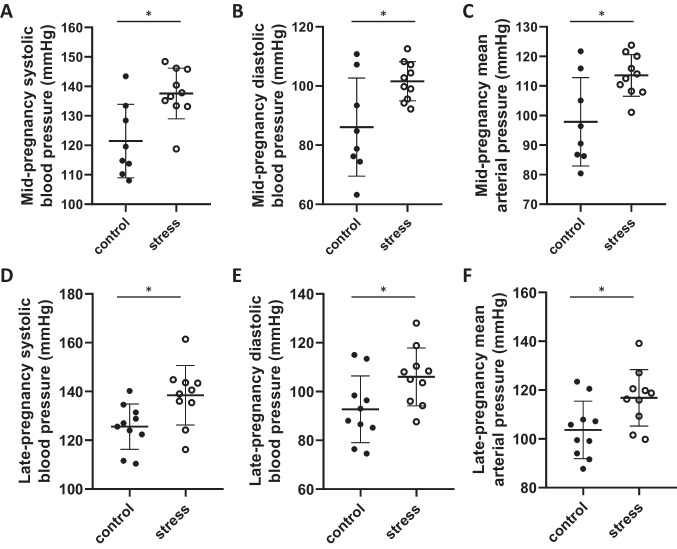
Blood pressure during pregnancy. Mean (± SD) **A** systolic, **B** diastolic, and **C** mean arterial blood pressure at mid-pregnancy (GD 9.5–10.5, control *n* = 8, stress *n* = 10) and **A** systolic, **B** diastolic, and **C** mean arterial blood pressure at late pregnancy (GD 14.5–17.5, control *n* = 10, stress = 10). **A**, **D** Systolic, **B**, **E** diastolic, and **C**, **F** mean arterial blood pressure was elevated in stress exposed females throughout the duration of pregnancy. * denotes significant difference (p < 0.05)

Pre-gestational stress exposure increased systolic (*p* < 0.01, *T* = 3.2, *F* = 2.1, df = 18, Fig. 2A), diastolic (*p* = 0.02, *T* = 2.7, *F* = 6.3, df = 18, Fig. 1B), and mean arterial (*p* = 0.01, *T* = 2.9, *F* = 4.5, df = 18, Fig. 1C) blood pressure at mid pregnancy (GD 9.5–10.5) compared to control females. Elevated blood pressure persisted into late pregnancy (GD 14.5–17.5) with systolic (*p* = 0.02, *T* = 2.7, *F* = 1.7, df = 18, Fig. 2D), diastolic (*p* = 0.03, *T* = 2.3, *F* = 1.3, df = 18, Fig. 2E), and mean arterial (*p* = 0.02, *T* = 2.5, *F* = 1.2, df = 18, Fig. 2F) blood pressure being significantly elevated in stress-exposed females compared to controls.

In the first week of pregnancy, stress-exposed females gained significantly less weight (*p* < 0.01, *T* =  − 4.05, *F* = 16.9, df = 15, Table 2) compared to control females. However, there was no difference in percentage weight gain in the remaining weeks of pregnancy between groups (Table 2). At the end of pregnancy (GD 17.5), stress exposed females had significantly larger hearts (*p* = 0.02, *T* = 2.6, *F* = 1.5, df = 15, Table 2) compared to control females. Female age at conception was not a significant covariate for any measure of early pregnancy weight gain, blood pressure, or heart weight (*p* > 0.05).

**Table 2:** Maternal and litter characteristics

	Control (*n* = 10)	Stress (*n* = 7)
Rebreeding attempts	1.4 ± 1.0	2.3 ± 0.7 *
Pregnancy weight gain (%)		
Week 1	16.7 ± 4.1	4.9 ± 2.5 *
Week 2	6.9 ± 4.5	9.7 ± 3.6
Week 3	28.3 ± 6.1	33.6 ± 8.5
Week 4	9.9 ± 4.9	10.3 ± 6.6
Maternal heart weight (grams)	0.15 ± 0.01	0.18 ± 0.02 *
Litter size	7 ± 1	5 ± 2
Total resorptions	1 ± 0.7	3 ± 1.3 *
Pup weight (grams)	0.74 ± 0.1	0.76 ± 0.2
Placental weight (grams)	0.094 ± 0.01	0.092 ± 0.01*
Pup:placenta weight ratio	8.0 ± 1.3	8.1 ± 1.4
Decidual depth (µm)	253 ± 68	164 ± 31 *
Decidual/total placental depth	0.11 ± 0.03	0.07 ± 0.01 *
Junctional zone depth (µm)	433 ± 130	477 ± 60
Junctional zone/total placental depth	0.18 ± 0.05	0.20 ± 0.02
Labyrinth depth (µm)	1622 ± 65	1699 ± 209
Labyrinth/total placental depth	0.69 ± 0.02	0.71 ± 0.07

#### Pre-pregnancy Stress-Induced Pregnancy Loss and Thinner Maternal Decidua

Stress-exposure significantly impaired pregnancy maintenance and reproductive success, with 64% of stress-exposed females needing to be rebred after the first breeding attempt compared to 25% of controls (*p* = 0.02). At the second attempt, 46% of stress-exposed females required rebreeding compared to 0% of control females (*p* = 0.02). This resulted in a greater number of rebreeding attempts overall in stress-exposed females relative to controls when comparing rebred females (*p* = 0.04, Table 2, *T* = 2.2, *F* = 2.1, df = 15).

Stress-exposed females had smaller litter sizes compared to control females, however this result was not statistically different (*p* = 0.1, Table 2). However, females exposed to CUS had a greater number of fetal resorptions at GD17.5 (*p* = 0.01, *T* = 2.1, *F* = 10.5, df = 15, Table 2) compared to control females. Further, after controlling for litter size, placentas collected from stress exposed females weighed significantly less than those isolated from control females (*p* < 0.01, *T* = 0.3, *F* = 1.4, df = 15, Table 2). After controlling for litter size, there was no difference in pup weight (*p* = 0.9, Table 2) or pup:placenta ratio (*p* = 0.4, Table 2) between groups.

Because of the significant impact of stress exposure on pregnancy observed in the current work, we investigated placental histology in a subset of females to study the effect of stress-related disorders on placental health. Stress-exposed females had significantly reduced depth of the decidua (*p* = 0.01, *T* = 2.9, *F* = 4.7, df = 15, Table 2) compared to control females. This effect persisted after controlling for overall placental depth, with a significant reduction in decidua/total placental depth in stress-exposed females (*p* = 0.01, *T* = 3.1, *F* = 3.9, df = 15, Table 2) compared to controls. There was no effect on depth of the junctional zone or labyrinth regions of the placenta between CUS and control females (*p* > 0.1, Table 2). Female age at conception was not a significant covariate for any measure of placental development (*p* > 0.05).

#### Pre-pregnancy Stress Blunted Endothelial-Dependent and -Independent Vascular Function During Pregnancy

**Fig. 3:**
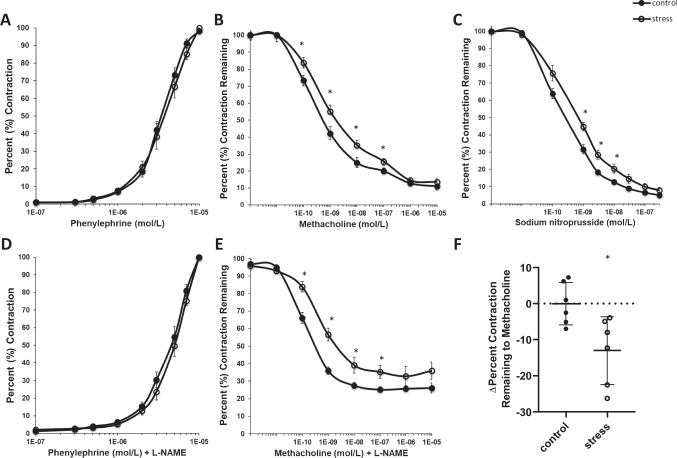
Endothelial function during late pregnancy. Mean (± SEM) percent **A**,**D** contraction and **B**, **C**, **E** relaxation responses from isometric wire myography of mesenteric arteries during late pregnancy (gestation day 17.5). **F** Mean (± SD) change in relaxation response to methacholine in the presence of L-NAME and methacholine. All values before 1E − 10 mol/L display pre-constrictive values. * denotes significant difference (*p* < 0.05) (control n = 10, stress *n* = 7)

During late pregnancy (GD 17.5), mesenteric arteries isolated from stress^−^exposed dams exhibited no difference in contractile response to phenylephrine (*p* ≥ 0.08, *T* < 2.1, *F* ≤ 1.3, df = 15, Fig. 3A) when compared to artery response of control dams (PE EC50 3.7 × 10^−6^ ± 1.4 × 10^−6^ vs. 3.6 × 10^−6^ ± 9.7 × 10^−7^ mol/L respectively, *p* = 0.69). Mesenteric artery relaxation to methacholine was significantly blunted in dams exposed to chronic perinatal stress (*p* ≤ 0.03, *T* < 2.6, *F* ≤ 1.6, df = 15, Fig. 3B) compared to control dams, highlighting impaired endothelial-dependent relaxation in a model of perinatal stress-related disorders. During late pregnancy, stress-exposed dams also exhibited a blunted relaxation response to sodium nitroprusside (*p* ≤ 0.03, *T* < 3.6, *F* ≤ 3.0, df = 15, Fig. 3C) compared to control dams, suggesting vascular dysfunction which also has an endothelial-independent component.

To further investigate the effect of CUS on the NO signaling pathway, we assessed contractile response to phenylephrine and relaxation response to methacholine in the presence of the NO synthase (NOS) inhibitor L-NAME. During late pregnancy, there was no difference in the contractile response of mesenteric arteries isolated from stress-exposed or control dams in the presence of L-NAME (*p* ≥ 0.09, Fig. 3D). However, stress-exposed dams exhibited a significantly blunted relaxation response to methacholine in the presence of L-NAME (*p* ≤ 0.01, *T* < 5.1, *F* ≤ 4.3, df = 15, Fig. 3E) compared to control dams, indicating endothelial-dependent dysfunction partially attributable to NO mechanisms. Change in relaxation response to methacholine and methacholine in the presence of L-NAME was lower in stress-exposed dams compared to control females (*p* ≤ 0.03, *T* < 3.0, *F* ≤ 1.7, df = 15, Fig. 3F), suggesting impaired NO-dependent relaxation mechanisms in this model of perinatal stress-related mood disorders during late pregnancy. Female age at conception was not a significant covariate for any measure of vascular function during pregnancy (*p* > 0.05).

### Postpartum

#### Stress Exposure Altered Maternal Caregiving Behaviors and Induced Persistent Anxiety-Like Behavior

Exposure to chronic unpredictable stress prior to pregnancy increased the amount of time females spent off their nests from postpartum day 1 to 6 (*p* = 0.04, *T* = 2.1, *F* = 1.1, df = 14, Table 3) compared to control females. While stress exposure also decreased nest building and licking/grooming behaviors (*p* = 0.1, Table 3), these behaviors were not significantly different when compared to control females. There was also no significant difference in any measure of maternal caregiving behavior bouts (*p* > 0.09, Table 3) or self-grooming behavior (*p* = 0.1, Table 3) between groups.

**Table 3:** Postpartum maternal behavior

	Control (*n* = 7)	Stress (*n* = 9)
Maternal caregiving time (seconds)		
On nest	2110.7 ± 336.1	2084.0 ± 940.8
Off nest	716.1 ± 444.7	1162.3 ± 420.2 *
Nest building	118.0 ± 109.4	86.7 ± 71.2
Licking/grooming	281.7 ± 256.1	113.8 ± 107.8
Self-grooming	32.9 ± 18.2	104.8 ± 102.2
Total time	2543.3 ± 1067.1	2389.4 ± 373.5
Maternal caregiving (bouts)		
On nest	6.7 ± 3.3	8.4 ± 2.4
Off nest	4.7 ± 3.3	7.0 ± 3.1
Nest building	4.0 ± 2.3	3.6 ± 2.5
Licking/grooming	5.9 ± 3.7	4.6 ± 4.8
Self-grooming	1.3 ± 1.6	3.8 ± 3.4
Total bouts	17.9 ± 10.6	20.5 ± 7.5
Maternal open field		
Time to first movement (seconds)	3.9 ± 2.3	5.1 ± 3.8
Peripheral entries (bouts)	7.9 ± 5.4	3.7 ± 4.8
Bouts freezing (bouts)	11.0 ± 2.9	27.1 ± 8.7 *
Bouts rearing (bouts)	14.9 ± 11.5	4.4 ± 3.6 *
Central zone entries (bouts)	7.9 ± 5.4	3.7 ± 4.5

**Fig. 4:**
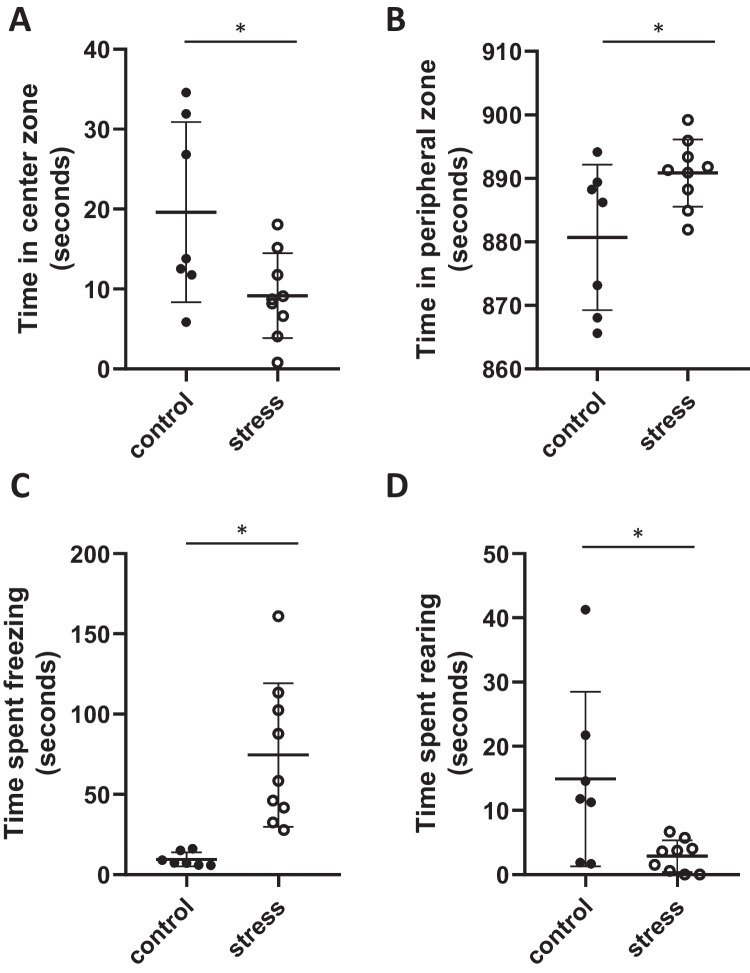
Postpartum open field behavior. Mean (± SD) time spent **A** in the center zone, **B** in the peripheral zone, **C** freezing, and **D** rearing in the open field arena. Stress exposure decreased time **A** in the center zone and **D** rearing behavior and increased time **B** in the peripheral zone and **C** time freezing when compared to control females. * denotes significant difference (*p* < 0.05) (control *n* = 7, stress *n* = 9)

At 1 month postpartum, exposure to pre-pregnancy stress significantly decreased time spent in the center zone of the open field arena (*p* = 0.02, *T* = 2.5, *F* = 4.5, df = 14, Fig. 4A), and increased time spent in the peripheral zone (*p* = 0.03, *T* = 2.4, *F* = 4.7, df = 14, Fig. 4B), compared to control females. Females exposed to stress prior to pregnancy also spent significantly more time freezing in the open field test (*p* < 0.01, T = 3.8, *F* = 4.7, df = 14, Fig. 4C), and displayed an increased number of freezing bouts (*p* < 0.01, *T* = 4.6, *F* = 8.9, df = 14, Table 3) compared to control females postpartum. Stress exposure decreased time spent rearing (exploring) in the open field area (*p* = 0.02, *T* = 2.6, *F* = 4.7, df = 14, Fig. 4D), and bouts rearing (*p* < 0.01, *T* = 2.6, *F* = 10.1, df = 14, Table 3), compared to control females. There was no effect of pre-pregnancy stress exposure on time to first movement in the open field test (*p* = 0.4, Table 3). There were also no differences in the number of entries into the peripheral or center zone of the arena between groups postpartum (Table 3). Female age at conception did not influence maternal behavior postpartum (*p* > 0.05).

#### Stress Exposure Had no Effect on Postpartum Body or Heart Weight, but Resulted in Sustained Higher Blood Pressure

**Fig. 5:**
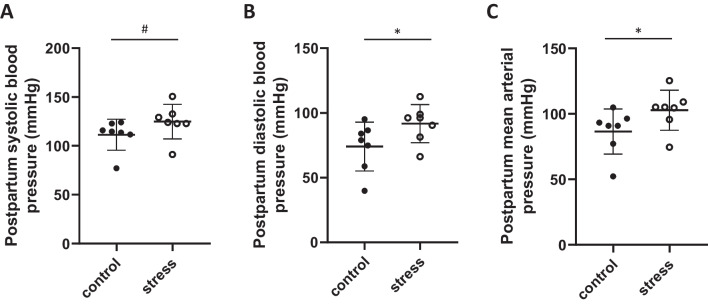
Postpartum blood pressure. Mean (± SD) **A** systolic, **B** diastolic, and **C** mean arterial blood pressure at 1 month postpartum. **A** Systolic blood pressure tended to be higher in stress exposed females (*p* = 0.05). **B** Diastolic and **C** mean arterial blood pressure was significantly elevated in stress exposed females when compared to control females at 1 month postpartum. * denotes significant difference (*p* < 0.05) (control *n* = 7, stress *n* = 7)

In the postpartum period, there was no significant difference in body weight at postpartum week 1, 2, 3 or 4 (*p* > 0.4, Table 4) or maternal heart weight (*p* = 0.2, Table 4) when comparing stress-exposed and control females. Diastolic (*p* = 0.03, T = 2.3, *F* = 2.8, df = 12, Fig. 5B) and mean arterial (*p* = 0.03, *T* = 2.4, *F* = 2.4, df = 12, Fig. 5C) blood pressure was elevated in pre-pregnancy stress exposed females compared to control females at 1 month postpartum. While systolic blood pressure tended to be higher in stress-exposed females compared to control females (*p* = 0.05, Fig. 5A), this was not statistically significant. Female age at conception did not influence postpartum blood pressure measures (*p* > 0.05).

**Table 4:** Maternal postpartum characteristics

	Control (*n* = 7)	Stress (*n* = 10)
Postpartum weight		
Week 1	31.6 ± 2.5	30.7 ± 3.3
Week 2	31.4 ± 1.9	32.2 ± 2.8
Week 3	30.3 ± 2.0	30.3 ± 3.0
Week 4	27.6 ± 3.1	27.6 ± 2.9
Maternal heart weight (g)	0.16 ± 0.02	0.17 ± 0.02

#### Vascular Dysfunction Persists in Stress-Exposed Females Postpartum

**Fig. 6:**
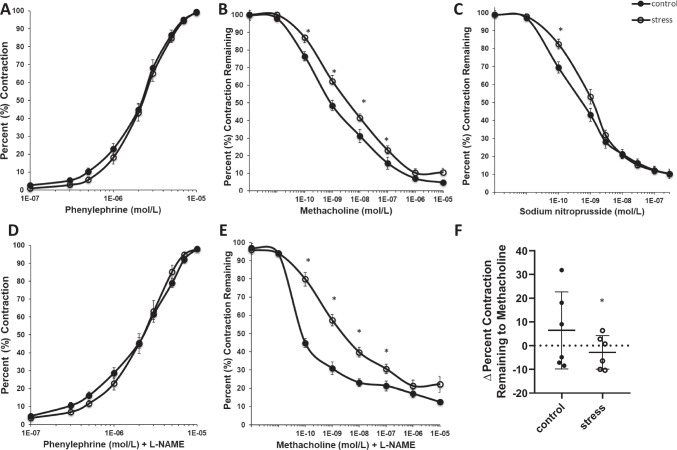
Endothelial function at 1 month postpartum. Mean (± SEM) percent **A**, **D** contraction and **B**, **C**, **E** relaxation responses from isometric wire myography of mesenteric arteries at one month postpartum. **F** Mean (± SD) change in relaxation response to methacholine in the presence of L-NAME and methacholine. All values before 1E − 10 mol/L display pre-constrictive values. * denotes significant difference (*p* < 0.05). (control *n* = 7, stress *n* = 10)

At 1 month postpartum, similar to observations during pregnancy, there was no difference in contractile response to phenylephrine (*p* > 0.08, Fig. 6A) between groups. Pre-pregnancy stress exposure significantly blunted relaxation response to methacholine (*p* ≤ 0.02, *T* ≤ 3.7, *F* ≤ 1.9, df = 15, Fig. 6B) when compared to control females, suggesting persistent endothelial-dependent dysfunction at 1 month postpartum. Stress exposure blunted relaxation response to sodium nitroprusside at the smallest dose (*p* = 0.02, *T* = 2.4, *F* = 2.4, df = 15, Fig. 6C), suggesting modest endothelial-independent dysfunction persisting postpartum.

Following NOS inhibition by L-NAME pre-incubation, mesenteric arteries isolated from stress exposed dams exhibited no difference in contractile response to phenylephrine (*p* ≥ 0.09, Fig. 6D) when compared to control dams postpartum. Stress-exposed dams exhibited blunted relaxation response to methacholine in the presence of L-NAME (*p* ≤ 0.02, *T* ≤ 7.6, *F* ≤ 2.9, df = 15, Fig. 6E) compared to control dams, highlighting endothelial-dependent dysfunction partially attributable to NO mechanisms. This is supported by a lower change in delta relaxation response to methacholine and methacholine in the presence of L-NAME in stress-exposed females (*p* ≤ 0.04, *T* ≤ 5.2, *F* ≤ 1.6, df = 15, Fig. 6F) compared to controls postpartum. Female age at conception did not influence postpartum measures of vascular function (*p* > 0.05).

#### Pre-pregnancy Stress Exposure Led to Lower Offspring Body Weight and Larger Heart to Body Weight Ratio at 1 Month Postpartum

At 1 month postpartum, after controlling for litter size, offspring of dams exposed to pre-pregnancy stress (*n* = 33) weighed less than offspring from control dams (*p* < 0.01, Table 5, *n* = 28). This decrease in weight gain was observed in both female (*p* = 0.04, Table 5, *n* = 12) and male offspring (*p* < 0.01, Table 5, *n* = 21) of stress-exposed dams compared to control female (*n* = 14) and male offspring (*n* = 14). While there was no difference in heart weight in females or males when comparing stress (*n* = 33) and control offspring groups (*n* = 28), there was a significant difference in heart to body weight ratio. At 1 month postpartum, after controlling for litter size, offspring of dams exposed to pre-pregnancy stress (*n* = 33) had larger heart to body weight ratios than offspring from control dams (*p* < 0.01, Table 5, *n* = 28). However, this appears due to stress exposure in males (*p* = *p* < 0.01, Table 5) with no effect in female stress-exposed offspring (Table 5).

**Table 5:** Postpartum offspring characteristics

	**Control** **(*n* = 28)**	**Stress** **(*n* = 33)**
Offspring body weight (g)	16.6 ± 1.3	15.7 ± 2.6 *
Offspring heart weight (g)	0.10 ± 0.01	0.10 ± 0.02
Heart:body weight ratio	0.0062 ± 0.0006	0.0076 ± 0.0006 *
	**Control** **(*n* = 14)**	**Stress** **(*n* = 12)**
Female body weight (g)	16.0 ± 0.9	15.6 ± 1.5 *
Female heart weight (g)	0.10 ± 0.01	0.09 ± 0.01
Female heart:body ratio	0.0064 ± 0.0009	0.0063 ± 0.0008
	**Control** **(*n* = 14)**	**Stress** **(*n* = 21)**
Male body weight (g)	17.2 ± 1.3	15.8 ± 3.0 *
Male heart weight (g)	0.10 ± 0.01	0.11 ± 0.02
Male heart:body ratio	0.0059 ± 0.0007	0.0068 ± 0.0008 *

Group/group size are indicated in bold font

## Discussion

The current work highlights persistent effects of chronic unpredictable stress and stress-related disorders during pregnancy and post-partum on maternal vascular health. Main findings show that pre-pregnancy stress induced an anxious-like phenotype in open field testing. Further, pre-pregnancy CUS led to increased systolic, diastolic, and mean arterial pressure and resulted in impaired ex vivo vascular function during pregnancy. Poor maternal vascular health following perinatal stress was supported by disruptions in pregnancy success and changes in placental histology. Importantly, this model of pre-pregnancy stress-related disorders resulted in impaired maternal health which persisted into the postpartum period, suggesting that pregnancies subjected to stress-related disorders may contribute to impaired vascular health later in life.

### Chronic Unpredictable Stress and Perinatal Behavior

In the current study, exposure to chronic unpredictable stress prior to pregnancy resulted in a more anxious-like phenotype. CUS exposure increased the amount of time spent in the periphery and freezing behavior during the open field test, suggesting that stress exposure even prior to pregnancy increases anxiety-like behavior. This supports previous research, documenting that stress paradigms induce similar behaviors. Exposure to chronic stress has been shown to increase freezing, time spent in the periphery during open field testing [17, 18], and immobility during the force swim test [31, 43] in rat dams. The anxious phenotype following perinatal CUS is also supported by the current study which showed a tendency for CUS exposed females to exhibit decreased time exploring the open field arena compared to control animals. Together, this supports that the CUS model is a well-documented model of both anxiety and depressive-like behavior, in addition to alterations in neuroendocrine measures [18, 27–30]. Further, findings from the current study extend previous work in rats to BALB/c mouse models, which traditionally display a more anxious phenotype than other mice [44]. Taken together, perinatal CUS in a BALB/c mouse model mimics stress-related disorders of pregnancy.

Current work shows that the impact of CUS exposure on behavior persists into the postpartum period, even when stress exposure occurs prior to pregnancy. CUS exposure decreased time spent in the center zone, time rearing, and increased time in the periphery and freezing bouts compared to non-exposed dams. This work supports previous research showing that stress during pregnancy increases immobility at the end of the postpartum period in rats [31] and increases depressive-like behavior in mice postpartum [45]. Stress prior to pregnancy has also been shown to increase freezing behavior and distance traveled in rat open field testing immediately prior to pregnancy, supporting increased anxiety-like behavior after CUS exposure [18]. The current work supports that stress exposure and behavioral phenotypes mimic stress-related disorders, and that these phenotypes persist postpartum. Together, this work confirms that CUS exposure in BALB/c mice even prior to pregnancy serves as a robust and persistent model of stress-related disorders such as anxiety and depression.

Much of the published work assessing CUS and perinatal maternal outcomes has focused on rat models, which as a collective model organism have been shown to display clear, observable changes in maternal behaviors [18, 43]. While some have suggested that stress, particularly gestational stress, can alter maternal behavior and thus can impact offspring phenotype [27, 32, 43], this alteration in maternal behavior still supports clinical changes in perinatal maternal behavior and offspring interactions. In the present study, only minimal changes in maternal behavior were observed. CUS exposed dams displayed increased time spent off the nest. However, stressed dams displayed no differences in licking/grooming, nest building or self-grooming time compared to control animals. Differences between previous and current work suggest that the CUS BALB/c mouse model may be a reliable method of studying perinatal stress-related disorders, with minimal impact on maternal behaviors.

### Stress Induced Pregnancy Loss and Impaired Placental Development

In the current study, a model of stress-related disorders decreased reproductive success with stress exposed dams being significantly less likely to maintain pregnancy at the first and second breeding attempt. This supports previous research in rat models of CUS which document poor reproductive success and maintenance of pregnancy [18]. Findings in animal models mimic what is observed in the clinical setting, with stress and related disorders decreasing pregnancy success, even when stress exposure occurs prior to gestation [46]. Although many mechanisms have been suggested for the association between stress and pregnancy loss, the exact cause remains unclear. It is important to note that the current study did not assess the stage of estrus prior to breeding. Regardless, significant differences in breeding and maintenance of pregnancy between stress and control animals suggest an impact of stress and stress-related disorders on reproductive success.

In the current study, stress-exposure significantly reduced depth of the decidua compared to non-stressed controls. This finding suggests that stress may alter development of the uterine decidual layer, impacting placental development and pregnancy maintenance. This is supported by the observed increase in fetal resorptions and decrease in placental weight at gestation day 17.5 in stress exposed dams compared to control females. More work is needed to understand how stress, even prior to pregnancy, can impact development of the placenta and pregnancy success.

Despite stress-induced changes in pregnancy success and placental development, stress-exposed dams did not have statistically different litter sizes compared to control females. Further, there was no difference in maternal weight throughout pregnancy or pup weight at the end of pregnancy between groups. This finding suggests that while stress exposure is able to induce a more anxious-like phenotype, the model does not necessarily induce poor health or nutritional status in dams prior to pregnancy, which would contribute to poor vascular outcome.

### Pre-pregnancy Stress and Maternal Vascular Health During Pregnancy

In the current study, exposure to pre-pregnancy stress led to elevated systolic, diastolic, and mean arterial pressure during pregnancy. This outcome was evident during mid-pregnancy, at gestation day 9.5–10.5, and continued throughout late pregnancy at gestation day 14.5–17.5. Previous animal work outside of pregnancy has shown that exposure to chronic stress can increase systolic blood pressure and angiotensin receptor expression in rats [47] and alter blood pressure variability in mice [48]. Additional research has shown similar outcomes during the perinatal period. Exposure to chronic stress from gestation day 7 to 14 has been shown to increase blood pressure, vasomotility, and proteinuria and lower endothelium-derived relaxing factor release in pregnant rats at gestation day 20 [49]. Our findings extend previous work and suggest that stress even prior to pregnancy is capable of inducing an impaired vascular phenotype during pregnancy. Further, the observed increase in blood pressure continued throughout pregnancy, suggesting that the vascular dysfunction observed in a model of stress-related disorders can persist throughout the perinatal period. Taken together, this supports that stress, even prior to pregnancy, can induce maternal vascular dysfunction, which may prime individuals for poor perinatal vascular health.

Stress-induced changes in maternal vascular health are further supported by changes in endothelial-dependent and -independent vascular function observed during pregnancy. Ex vivo wire myography demonstrated a blunted relaxation response of the mesenteric arteries of stress exposed dams compared to control dams when exposed to methacholine, supporting endothelial-dependent dysfunction resulting from perinatal stress-related disorders. Further, stress-exposure blunted relaxation response to sodium nitroprusside, suggesting an additional endothelial-independent component downstream of NO production. Outside of pregnancy, chronic stress has been shown to contribute to poor vascular health via altered heart rate variability and blunted endothelial-dependent relaxation in rats [36, 50]. Chronic pre-gestational stress results in poor peripartum cardiac health via left ventricular systolic dysfunction in dams and cardiac abnormalities in rat offspring [34], although underlying mechanisms remain unclear. The current work provides supporting evidence that stress and related disorders can contribute to changes in vascular function during pregnancy, which may contribute to increased risk of vascular syndromes during pregnancy. This is supported by our previous work, which shows comparable vascular dysfunction in a mouse model of preeclampsia [39], mimicking the impaired maternal vascular health observed in the current study. This further supports the association between stress-related and vascular syndromes during pregnancy.

The current work provides evidence that the nitric oxide signaling pathway may be contributing to changes in vascular function in stress-exposed dams. During pregnancy, pre-pregnancy stress-exposed dams exhibited a significantly blunted relaxation response to methacholine in the presence of the nitric oxide synthase inhibitor L-NAME compared to control dams. These findings suggest that endothelial-dependent dysfunction may be partially attributable to NO mechanisms. The NO pathway is critical for vascular homeostasis during pregnancy. Nitric oxide is critical for implantation, vascular remodeling, placental development, fetoplacental perfusion, and embryonic development [51]. Further, the NO pathway has been implicated in stress-related mood disorders during pregnancy. Specifically, stress-related major depressive disorder during pregnancy is associated with lower levels of L-arginine, NO’s precursor, across pregnancy compared to pregnant women without stress-related mood disorders [52]. The current study provides supporting evidence that NO mechanisms, which are related to poor vascular health outside of the perinatal period, may also be related to pregnancy-specific vascular dysfunction in individuals with stress-related disorders. More work is needed to understand how changes in NO and NO signaling contribute to poor maternal vascular health and pregnancy success.

### Stress and Vascular Health Postpartum

In the current study, stress induced changes to blood pressure and vascular function were sustained in the postpartum period. Specifically, pre-pregnancy stress resulted in persistent elevated systolic, diastolic, and mean arterial pressure at 1 month postpartum compared to non-stressed controls. Although the increase in pregnancy heart weight of stressed dams did not persist postpartum, the current study did not assess specific morphologies associated with cardiac dysfunction. Previous work has shown that stress exposure prior to pregnancy results in left ventricular systolic dysfunction in dams and cardiac abnormalities in rodent offspring [34]. Together, these findings suggest that perinatal stress and related disorders may prime individuals for future cardiovascular disease.

In further support of persistent stress-induced vascular dysfunction, the current study showed stress-exposed females had persistent changes in ex-vivo vascular function compared to control dams. At 1 month postpartum, pre-pregnancy stress exposure significantly blunted relaxation response to methacholine and sodium nitroprusside, suggesting long term endothelial-dependent and independent dysfunction in a maternal model of stress-related disorders. This supports additional work outside of pregnancy linking chronic stress exposure to poor vascular health [53], and extends this work to the perinatal period. Further, similar to the observed patterns in vascular function during pregnancy, exposure to pre-pregnancy stress decreased relaxation response in the presence of L-NAME suggesting vascular dysfunction partially attributable to NO mechanisms. In the current work, mice were assessed at 28 days postpartum which is equivalent to approximately 3 years postpartum in humans [54]. Due to the link between perinatal vascular health and long-term cardiovascular risk, these vascular changes postpartum suggest that stress and related disorders even prior to pregnancy can affect future vascular disease. However, more work is needed to investigate the impact of CUS on long-term maternal cardiovascular health.

In the current study, the effects of stress exposure appear transgenerational and contribute to differences in offspring phenotype. Stress exposure decreased offspring body weight and increased heart:body weight ratio. Stress exposure prior to pregnancy has previously been shown to induce cardiac abnormalities in rodent offspring [29]. Together, this suggests that not only does perinatal stress prime poor maternal vascular health but can also negatively influence the development of offspring cardiovascular health. Similar to previous work, these data suggest that male offspring in particular may be more susceptible to the impact of pre-gestational stress exposure. Future work is needed to understand the long-term impact of pre-gestational stress on offspring cardiovascular function.

## Conclusions and Future Work

The current study has strengths and limitations. These data suggests that pre-pregnancy stress in a BALB/c mouse model is a useful model to investigate the effects of stress on maternal vascular health during pregnancy and postpartum. The study brings to light that stress, even prior to pregnancy, may prime individuals for poor vascular health during pregnancy which extends into the postpartum period. While we have observed significant differences in ex vivo vascular function using wire myography, we are not able to fully account for all possible vascular changes that may contribute to these data including possible changes in vascular tone. Mouse mesenteric arteries present unique challenges including a loss of tone ex vivo, which may have impacted the current findings. Future work will need to more fully investigate changes to ex-vivo vascular tone in mesenteric arteries, along with the mechanisms and effects of stress on vascular function in this model of pre-pregnancy stress. In addition, more work is needed to characterize the mechanisms of stress-induced changes in placental morphology and changes in vascular function. Further, although the current study assessed postpartum mice outcomes at 28 days, which is approximately 3 years postpartum in humans, additional studies assessing even longer time points after pregnancy might also help characterize the impact of stress on future cardiovascular disease. Additionally, as this work and clinical work has shown that stress exposure can delay time to successful pregnancy, future studies characterizing pregnancy success, maintenance, and age are warranted. Future work investigating the impact of CUS exposure on maternal health prior to pregnancy may also help better categorize the trajectory of vascular health.

In conclusion, our findings suggest stress exposure and stress-related disorders can induce poor maternal vascular health during pregnancy. Further, these effects can extend to the postpartum period, and suggest that pregnancy may serve as a unique time to understand individuals with challenged vascular health who may be at increased risk for cardiovascular disorders in later life. Taken together, this work suggests stress can directly contribute to poor maternal vascular health, perinatal comorbidities, and long-term cardiovascular disease. More work is needed to understand specific mechanisms contributing to maternal vascular dysfunction and how these alter maternal health during pregnancy and postpartum to promote maternal health later in life.

## Data Availability

The data that support the findings of this study are available from the corresponding author, MGO, upon reasonable request.

## References

[1] Silverman ME, Reichenberg A, Savitz DA, Cnattingius S, Lichtenstein P, Hultman CM (2017). The risk factors for postpartum depression: a population-based study. Depress Anxiety.

[2] Almond P (2009). Postnatal depression: a global public health perspective. Perspect Public Health.

[3] Leung BM, Kaplan BJ (2009). Perinatal depression: prevalence, risks, and the nutrition link–a review of the literature. J Am Diet Assoc.

[4] Pawluski JL, Lonstein JS, Fleming AS (2017). The Neurobiology of postpartum anxiety and depression. Trends Neurosci.

[5] Stepowicz A, Wencka B, Bieńkiewicz J, Horzelski W, Grzesiak M. Stress and anxiety levels in pregnant and post-partum women during the COVID-19 pandemic. Int J Environ Res Public Health. 2020;17(24):9450. 10.3390/ijerph17249450 PMC7766953 33348568

[6] Lebel C, MacKinnon A, Bagshawe M, Tomfohr-Madsen L, Giesbrecht G (2020). Elevated depression and anxiety symptoms among pregnant individuals during the COVID-19 pandemic. J Affect Disord.

[7] Moyer CA, Compton SD, Kaselitz E, Muzik M (2020). Pregnancy-related anxiety during COVID-19: a nationwide survey of 2740 pregnant women. Archives of women’s mental health.

[8] Laplante DP, Brunet A, King S (2016). The effects of maternal stress and illness during pregnancy on infant temperament: Project Ice Storm. Pediatr Res.

[9] Zhang W, Ham J, Li Q, Deyssenroth MA, Lambertini L, Huang Y (2020). Moderate prenatal stress may buffer the impact of Superstorm Sandy on placental genes: stress in Pregnancy (SIP) Study. PLoS ONE.

[10] Van den Bergh BRH, van den Heuvel MI, Lahti M, Braeken M, de Rooij SR, Entringer S (2020). Prenatal developmental origins of behavior and mental health: the influence of maternal stress in pregnancy. Neurosci Biobehav Rev.

[11] Gemmel M, Bögi E, Ragan C, Hazlett M, Dubovicky M, van den Hove DL, et al. Perinatal selective serotonin reuptake inhibitor medication (SSRI) effects on social behaviors, neurodevelopment and the epigenome. Neurosci Biobehav Rev. 2018;85:102–16. 10.1016/j.neubiorev.2017.04.023 28472631

[12] Gemmel M, Hazlett M, Bogi E, De Lacalle S, Hill LA, Kokras N (2017). Perinatal fluoxetine effects on social play, the HPA system, and hippocampal plasticity in pre-adolescent male and female rats: Interactions with pre-gestational maternal stress. Psychoneuroendocrinology.

[13] Gemmel M, Rayen I, Lotus T, van Donkelaar E, Steinbusch HW, De Lacalle S, et al. Developmental fluoxetine and prenatal stress effects on serotonin, dopamine, and synaptophysin density in the PFC and hippocampus of offspring at weaning. Dev Psychobiol. 2016;58(3):315–27. 10.1002/dev.21372 26477449

[14] Lackner HK, Moertl MG, Schmid-Zalaudek K, Lucovnik M, Weiss EM, Kolovetsiou-Kreiner V (2018). History of preeclampsia adds to the deleterious effect of chronic stress on the cardiac ability to flexibly adapt to challenge. Front Physiol.

[15] Strapasson MR, Ferreira CF, Ramos JGL (2018). Associations between postpartum depression and hypertensive disorders of pregnancy. Int J Gynaecol Obstet..

[16] Lancaster CA, Gold KJ, Flynn HA, Yoo H, Marcus SM, Davis MM (2010). Risk factors for depressive symptoms during pregnancy: a systematic review. Am J Obstet Gynecol.

[17] Gemmel M, Rayen I, van Donkelaar E, Loftus T, Steinbusch HW, Kokras N (2016). Gestational stress and fluoxetine treatment differentially affect plasticity, methylation and serotonin levels in the PFC and hippocampus of rat dams. Neuroscience.

[18] Gemmel M, Harmeyer D, Bogi E, Fillet M, Hill LA, Hammond GL (2018). Perinatal fluoxetine increases hippocampal neurogenesis and reverses the lasting effects of pre-gestational stress on serum corticosterone, but not on maternal behavior, in the rat dam. Behav Brain Res.

[19] Rosman L, Salmoirago-Blotcher E, Cahill J, Wuensch KL, Sears SF (2017). Depression and health behaviors in women with peripartum cardiomyopathy. Heart Lung.

[20] Jasper R, Skelding K (2018). Cardiovascular disease risk unmasked by pregnancy complications. Eur J Intern Med.

[21] Tooher J, Thornton C, Makris A, Ogle R, Korda A, Hennessy A (2017). All hypertensive disorders of pregnancy increase the risk of future cardiovascular disease. Hypertension.

[22] Armario A, Escorihuela RM, Nadal R (2008). Long-term neuroendocrine and behavioural effects of a single exposure to stress in adult animals. Neurosci Biobehav Rev.

[23] Belda X, Fuentes S, Nadal R, Armario A (2008). A single exposure to immobilization causes long-lasting pituitary-adrenal and behavioral sensitization to mild stressors. Horm Behav.

[24] Armario A, Marti O, Valles A, Dal-Zotto S, Ons S (2004). Long-term effects of a single exposure to immobilization on the hypothalamic-pituitary-adrenal axis: neurobiologic mechanisms. Ann N Y Acad Sci.

[25] Frisbee JC, Brooks SD, Stanley SC, d'Audiffret AC. An unpredictable chronic mild stress protocol for instigating depressive symptoms, behavioral changes and negative health outcomes in rodents. J Vis Exp. 2015;(106):53109. 10.3791/53109 PMC4692768 26650668

[26] Shoji H, Miyakawa T (2019). Increased depression-related behavior during the postpartum period in inbred BALB/c and C57BL/6 strains. Mol Brain.

[27] Champagne FA, Meaney MJ (2006). Stress during gestation alters postpartum maternal care and the development of the offspring in a rodent model. Biol Psychiatry.

[28] Darnaudery M, Dutriez I, Viltart O, Morley-Fletcher S, Maccari S (2004). Stress during gestation induces lasting effects on emotional reactivity of the dam rat. Behav Brain Res.

[29] Huang Y, Xu H, Li H, Yang H, Chen Y, Shi X (2012). Pre-gestational stress reduces the ratio of 5-HIAA to 5-HT and the expression of 5-HT1A receptor and serotonin transporter in the brain of foetal rat. BMC Neurosci.

[30] Van den Hove DL, Blanco CE, Aendekerk B, Desbonnet L, Bruschettini M, Steinbusch HP (2005). Prenatal restraint stress and long-term affective consequences. Dev Neurosci.

[31] O'Mahony SM, Myint AM, van den Hove D, Desbonnet L, Steinbusch H, Leonard BE (2006). Gestational stress leads to depressive-like behavioural and immunological changes in the rat. NeuroImmunoModulation.

[32] Smith JW, Seckl JR, Evans AT, Costall B, Smythe JW (2004). Gestational stress induces post-partum depression-like behaviour and alters maternal care in rats. Psychoneuroendocrinology.

[33] Stewart DE (2011). Clinical practice. Depression during pregnancy. N Engl J Med..

[34] Bouzinova EV, Norregaard R, Boedtkjer DM, Razgovorova IA, Moeller AM, Kudryavtseva O (2014). Association between endothelial dysfunction and depression-like symptoms in chronic mild stress model of depression. Psychosom Med.

[35] Cox BM, Alsawah F, McNeill PC, Galloway MP, Perrine SA (2011). Neurochemical, hormonal, and behavioral effects of chronic unpredictable stress in the rat. Behav Brain Res.

[36] Grippo AJ, Beltz TG, Johnson AK (2003). Behavioral and cardiovascular changes in the chronic mild stress model of depression. Physiol Behav.

[37] Hillerer KM, Neumann ID, Slattery DA (2012). From stress to postpartum mood and anxiety disorders: how chronic peripartum stress can impair maternal adaptations. Neuroendocrinology.

[38] Martin AL, Brown RE (2010). The lonely mouse: verification of a separation-induced model of depression in female mice. Behav Brain Res.

[39] Sutton EF, Gemmel M, Brands J, Gallaher MJ, Powers RW. Paternal deficiency of complement component C1q leads to a preeclampsia-like pregnancy in wild-type female mice and vascular adaptations postpartum. Am J Physiol Regul Integr Comp Physiol. 2020;318(6):R1047–57. 10.1152/ajpregu.00353.2019 32374620

[40] He MY, Wang G, Han SS, Jin Y, Li H, Wu X, et al. Nrf2 signalling and autophagy are involved in diabetes mellitus-induced defects in the development of mouse placenta. Open Biol. 2016;6(7):160064 10.1098/rsob.160064 PMC4967824 27383629

[41] Hu X, Li J, Zhang Q, Zheng L, Wang G, Zhang X (2016). Phosphoinositide 3-kinase (PI3K) subunit p110delta is essential for trophoblast cell differentiation and placental development in mouse. Sci Rep.

[42] Tal R, Shaish A, Barshack I, Polak-Charcon S, Afek A, Volkov A (2010). Effects of hypoxia-inducible factor-1alpha overexpression in pregnant mice: possible implications for preeclampsia and intrauterine growth restriction. Am J Pathol.

[43] Brummelte S, Galea LA (2010). Depression during pregnancy and postpartum: contribution of stress and ovarian hormones. Prog Neuropsychopharmacol Biol Psychiatry.

[44] Russo AM, Lawther AJ, Prior BM, Isbel L, Somers WG, Lesku JA (2019). Social approach, anxiety, and altered tryptophan hydroxylase 2 activity in juvenile BALB/c and C57BL/6J mice. Behav Brain Res.

[45] Misdrahi D, Pardon MC, Perez-Diaz F, Hanoun N, Cohen-Salmon C (2005). Prepartum chronic ultramild stress increases corticosterone and estradiol levels in gestating mice: implications for postpartum depressive disorders. Psychiatry Res.

[46] Frazier T, Hogue CJR, Bonney EA, Yount KM, Pearce BD (2018). Weathering the storm; a review of pre-pregnancy stress and risk of spontaneous abortion. Psychoneuroendocrinology.

[47] Bobrovskaya L, Beard D, Bondarenko E, Beig MI, Jobling P, Walker FR (2013). Does exposure to chronic stress influence blood pressure in rats?. Auton Neurosci.

[48] Farah VM, Joaquim LF, Bernatova I, Morris M (2004). Acute and chronic stress influence blood pressure variability in mice. Physiol Behav.

[49] Takiuti NH, Kahhale S, Zugaib M (2002). Stress in pregnancy: a new Wistar rat model for human preeclampsia. Am J Obstet Gynecol.

[50] Grippo AJ (2009). Mechanisms underlying altered mood and cardiovascular dysfunction: the value of neurobiological and behavioral research with animal models. Neurosci Biobehav Rev.

[51] Sutton EF, Gemmel M, Powers RW (2019). Nitric oxide signaling in pregnancy and preeclampsia. Nitric Oxide.

[52] Raw A, Gallaher M, Powers RW (2014). Arginine and asymmetric dimethylarginine in pregnant women with major depression. Psychosom Med.

[53] Golbidi S, Frisbee JC, Laher I (2015). Chronic stress impacts the cardiovascular system: animal models and clinical outcomes. Am J Physiol Heart Circ Physiol.

[54] Dutta SSP (2016). Men and mice: relating their ages. Life Sci.

